# Noninvasive diagnosis of chemotherapy induced liver injury by LiMAx test – two case reports and a review of the literature

**DOI:** 10.1186/s13104-015-1055-6

**Published:** 2015-03-26

**Authors:** Jan Bednarsch, Maximilian Jara, Johan Friso Lock, Maciej Malinowski, Johann Pratschke, Martin Stockmann

**Affiliations:** Department of General, Visceral and Transplantation Surgery, Charité University Hospital, Augustenburger Platz 1, 13353 Berlin, Germany

**Keywords:** LiMAx, ICG-PDR, Liver function, Chemotherapy induced liver injury, Methacetin

## Abstract

**Background:**

Chemotherapy-induced liver injury is a well-known phenomenon after neoadjuvant therapy of liver metastasis and contributes to postoperative morbidity and mortality. Still there is no suitable test available to reliably determine functional impairment and hepatic regeneration after chemotherapy.

**Case presentation:**

We report two cases of caucasian patients who underwent repeated liver function assessments using LiMAx (maximum liver function capacity), Indocyanine plasma disappearance rate and biochemical liver function parameters in the course of adjuvant oxaliplatin-based chemotherapy.

Both patients yielded a decrease from their initial liver function determined by LiMAx. Liver regeneration assessed functional recovery within 4 weeks in case of mild functional impairment after cessation of chemotherapy or within 8 weeks in case of major functional deterioration. Indocyanine plasma disappearance rate and biochemical parameters remained stable or without a clear trend in case of minor functional impairment. This is the first report using a dynamic liver function test to evaluate the impact and recovery from chemotherapy associated liver injury.

**Conclusions:**

The LiMAx test might be a sensitive tool to diagnose mild functional impairment after chemotherapy when standard liver function tests have remained within normal ranges and might be capable to assess the course of regeneration after chemotherapy. This could be useful to optimize individual chemotherapy-free interval before liver surgery can be carried out safely.

## Background

Chemotherapy-induced liver injury (CALI) after receiving potent chemotherapy is a considerable problem in patients undergoing partial liver resection since increased perioperative mortality and morbidity have been reported [1-3]. However patients presenting with multiple liver metastasis frequently require preoperative chemotherapy to downstage their initially unresectable disease [4-6]. In this context oxaliplatin-based chemotherapeutic regimes – e.g. FOLFOX or FOLFOXIRI – showed promising ability to downstage spread and size but also increased perioperative morbidity [3,7,8].

Whereas previous studies were based on the clinical course and histology of resected liver parenchyma after surgery, less is known about the impact on actual liver function and the course of regeneration after cessation of chemotherapy [9,10].

Clinical biochemistry usually fail to diagnose reduced liver function after neoadjuvant therapy as standard parameters remain within normal ranges [11]. The frequently applied indocyanine green plasma disappearance rate test (ICG-PDR) also showed conflicting results in predicting operative outcome after chemotherapy [7,12-15].

Concerning the chemotherapy-free interval from cessation of chemotherapy to a safe partial liver resection only statistical figures are known in the literature and suggest an interval of six to eight weeks [16]. There is evidence that vulnerability to especially oxaliplatin-based liver injury might be different between patients. Thus an individual time frame between chemotherapy and surgery based on individual liver function and regeneration might be superior to a fixed interval [17,18].

## Case presentation

Here we report two cases of patients that both received adjuvant oxaliplatin-based chemotherapy as a consequence of colorectal surgery due to adenocarcinoma. All patients underwent assessment of liver function using common biochemical liver tests, ICG-PDR and LiMAx (maximum liver function capacity) test before, directly after withdrawal as well as 4 and 8 weeks after cessation of chemotherapy. Both patients did not have additional regular drug intake during the study period and did not suffer from any marked comorbidities known to impair liver function.

**Figure 1:**
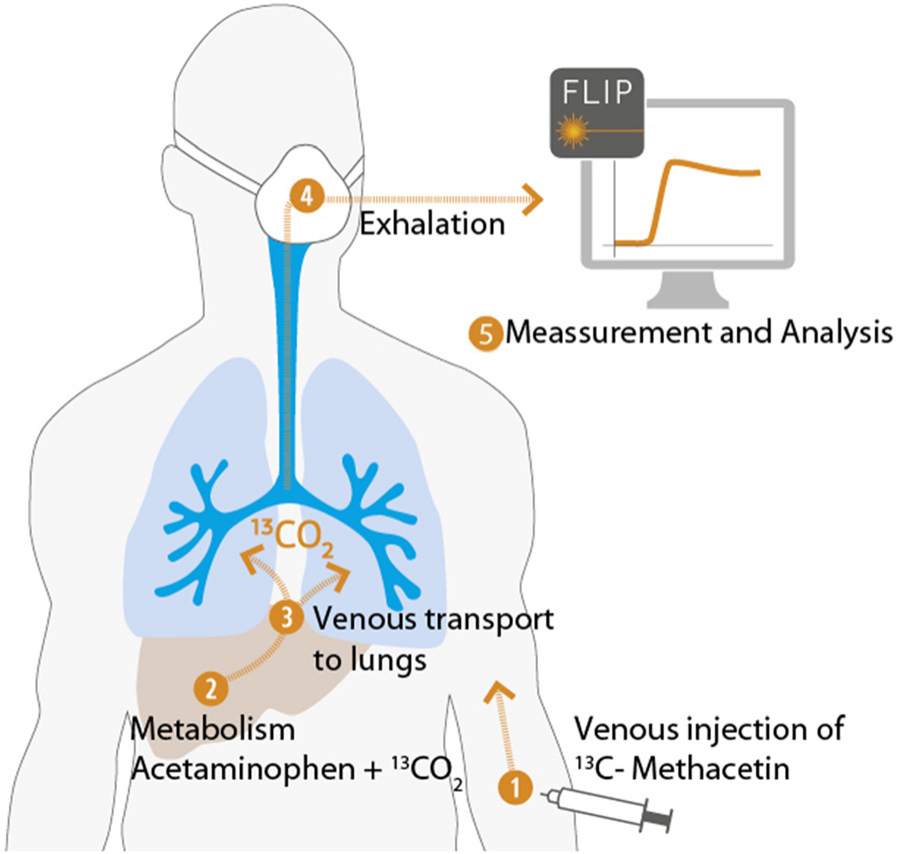
**Concept of the LiMAx test.** The figure is property of Humedics GmbH (Berlin, Germany), the company marketing the LiMAx test. The authors have the unrestricted permission to use the figure for this publication.

LiMAx (maximum liver function capacity) reflects the actual enzymatic liver function capacity. The test is based on a bodyweight-adjusted intravenous ^13^C-labeled methacetin bolus injection and continuous measurement of ^13^CO_2_/^12^CO_2_ concentration ratio using a special device (FLIP, Humedics GmbH, Berlin, Germany) as previously described by Stockmann and colleagues [19]. The LiMAx test (Figure 1) was developed and recently introduced into clinical routine in our department showing superior accuracy in diagnosing postoperative liver failure and early graft dysfunction after liver transplantation [19-21]. LiMAx values >315 μg/kg/h are considered normal.

Indocyanine green plasma disappearance rate (ICG-PDR) evaluates hepatic clearance and is suggested to provide additional information on liver function. The test is based on bodyweight-adjusted intravenous injection of ICG followed by pulse spectrophotometry to assess hepatic excretion via a special device (Dye Densitogram Analyzer DDG2001, Nihon Khoden, Tokyo, Japan). ICG-PDR is considered to be normal over 18%/min [22,23].

### Case 1

**Figure 2:**
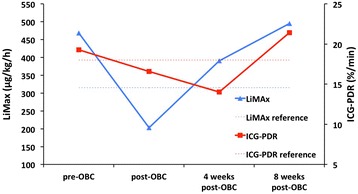
**Dynamic liver function assessment by LiMAx and Indocyanine green plasma disappearance rate in Case 1.** Patients’ liver function capacity measured by LiMAx was reduced by 56% after chemotherapy and ICG-PDR by 14%. Despite showing a different course of regeneration, both dynamic liver function tests indicate functional recovery within 8 weeks after cessation of chemotherapy. pre-Chemo – prior to chemotherapy, post-Chemo – after cessation of chemotherapy, 4 weeks – 4 weeks after cessation of chemotherapy, 8 weeks – 8 weeks after cessation of chemotherapy.

Case 1 reports a 56-year-old male caucasian patient that received 8 cycles of XELOX chemotherapy substituting 5-FU for the oral drug Xeloda. The patient started XELOX chemotherapy with a LiMAx value of 463 μg/kg/h and showed a decrease of functional liver capacity by 56% to 204 μg/kg/h after withdrawal of chemotherapy. ICG-PDR also declined from physiological 19.3%/min prior to chemotherapy to 16.6%/min (Figure 2). In terms of regeneration LiMAx indicates a continuous course regaining liver function after a chemotherapy-free interval of 8 weeks. In contrast ICG-PDR persisted to decrease until 4 weeks after cessation of chemotherapy. However it also restored its value by week 8. Biochemistry showed a peak of serum bilirubin directly after chemotherapy and a temporary decrease in butyrylcholinesterase (BChE) until 8 weeks after withdrawal of XELOX (Table 1).

**Table 1:** **Liver function assessment of case 1**

**Laboratory parameters**	**Reference range**	**pre-Chemo**	**post-Chemo**	**4 weeks**	**8 weeks**
LiMAx (μg/kg/h)	>315	468	203	390	495
ICG-PDR (%/min)	>18	19.3	16.6	14	21.4
ALT (U/L)	10 - 50	41.00	33.00	34.00	41.00
Bilirubin (mg/dL)	<1	0.6	2.15	1.08	1.03
INR	0,7 - 1,3	0.97	1.43	1.04	1.04
BChE (kU/L)	>5.3	6.47	3.88	4.97	7.11

pre-Chemo – prior to chemotherapy, post-Chemo – after cessation of chemotherapy, 4 weeks – 4 weeks after cessation of Chemotherapy, 8 weeks – 8 weeks after cessation of chemotherapy, LiMAx – Maximum liver function capacity, ICG-PDR, Indocyanine green plasma disappearance rate, ALT – Alanine transaminase, INR – international normalized ratio, BChE – Butyrylcholinesterase.

### Case 2

**Figure 3:**
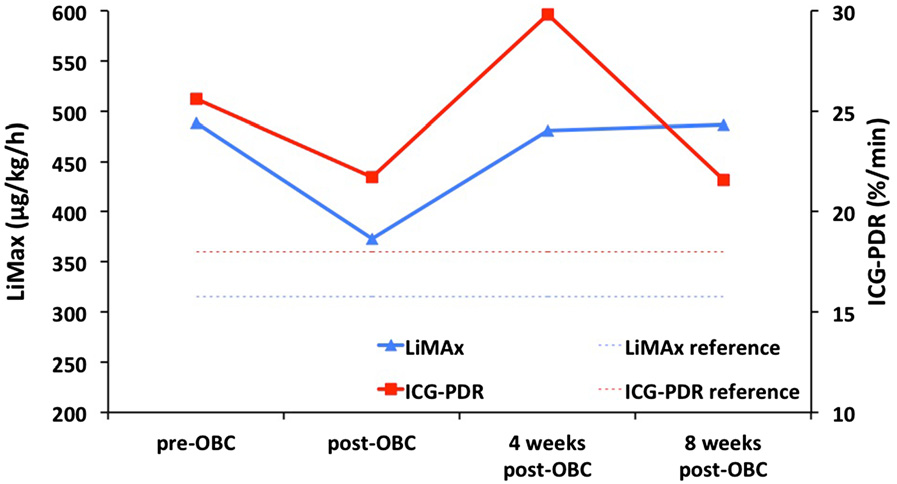
**Dynamic liver function assessment by LiMAx and Indocyanine green plasma disappearance rate in Case 2.** Patients’ liver function capacity measured by LiMAx was reduced by 24% after chemotherapy. Four weeks after cessation of FOLFOX the patient had already regained former liver function. ICG PDR showed no clear trend. pre-Chemo – prior to chemotherapy, post-Chemo – after cessation of chemotherapy, 4 weeks – 4 weeks after cessation of chemotherapy, 8 weeks – 8 weeks after cessation of chemotherapy.

Case 2 is a 68-year-old female caucasian patient that underwent 12 cycles of FOLFOX4 chemotherapy. This patient started chemotherapy with a LiMAx value of 488 μg/kg/h and showed a decrease of functional liver capacity by 24% to 373 μg/kg/h after withdrawal of chemotherapy. Four weeks after cessation of chemotherapy initial liver function was regained and remained constant during follow-up. ICG-PDR showed no clear trend and remained throughout all visits within the reference range (Figure 3). Biochemical liver parameters were also within the reference ranges on each visit (Table 2).

**Table 2:** **Liver function assessment of case 2**

**Laboratory parameters**	**Reference range**	**pre-Chemo**	**post-Chemo**	**4 weeks**	**8 weeks**
LiMAx (μg/kg/h)	>315	488	373	481	487
ICG-PDR (%/min)	>18	25.6	21.7	29.8	21.6
ALT (U/L)	7 - 35	13.00	26.00	17.00	21.00
Bilirubin (mg/dL)	<1	0.27	0.62	0.56	0.39
INR	0,7 - 1,3	0.95	1.12	0.95	1.02
BChE (kU/L)	>5.3	6.32	5.64	6.10	6.69

pre-Chemo – prior to chemotherapy, post-Chemo – after cessation of chemotherapy, 4 weeks – 4 weeks after cessation of Chemotherapy, 8 weeks – 8 weeks after cessation of chemotherapy, LiMAx – Maximum liver function capacity, ICG-PDR, Indocyanine green plasma disappearance rate, ALT – Alanine transaminase, INR – international normalized ratio, BChE – Butyrylcholinesterase.

## Discussion

To our knowledge this is the first report using quantitative liver function tests to assess deterioration and regeneration of liver function duo to chemotherapy in controlled prospective design with multiple testing. The reported patients may be of interest as on patient showed changes in common blood liver function tests indicating marked impairment of liver function whereas the other patient had no significant changes in standard liver function tests.

In accordance with this observation both dynamic liver function tests showed a major decrease in liver function reaching pathological values in case 1 in comparisons to case 2 where only a mild impairment of liver function could be measured with the LiMAx test. LiMAx indicates a defined course of regeneration in both cases but a delay in case 1. ICG-PDR seems to be a good indicator for regeneration in the case of major impairment of liver function (case 1), however ICG-PDR results in case 2 are inconclusive. This might be explained by general limitations of this testing procedure. ICG-PDR is strongly dependent on blood flow to the liver and the absence of cholestasis [24-26]. Since damage to liver sinusoids accompanied with sinusoidal obstruction is the basic pathological mechanism in oxaliplatin-based liver injury it is debatable whether ICG-PDR is a good indicator for liver function in this explicit clinical situation [9,27]. The usefulness of ICG-PDR after chemotherapy has already been questioned by Wakiya *et al*. [15].

Both LiMAx and ICG-PDR have shown superior prognostic relevance for perioperative mortality and morbidity in patients undergoing partial liver resection compared to standard liver function tests [19,20,28,29]. Clinical decision trees exist for both testing procedures allowing accurate risk stratification and prediction of postoperative liver failure. The presented cases support the hypothesis that regeneration of liver function has a defined course, which is accessible by means of dynamic liver functions tests which could be used to evaluate the optimal chemotherapy-free interval prior to partial liver resection using adequate decision algorithms [20,28]. In particular enzymatic based LiMAx test may also assess mild impairment of liver function – which can contribute to poorer postoperative outcome – providing additional information compared to standard blood borne liver function tests and ICG-PDR.

Certainly we present cases in patients without liver metastases and without indication for liver surgery. However our workgroup has already shown that the sole presence of liver metastasis which is usually advanced in patients that undergo neoadjuvant treatment can also impair liver function making it impossible to discriminate between reduction of liver function by chemotherapy or multiple metastasis in a proof of principle report [30].

## Conclusions

This is the first report using a dynamic liver function test in a controlled prospective setting to assess chemotherapy induced liver injury. It suggests an impairment of liver function, which might not be recognised by standard liver function tests and a defined course of regeneration after chemotherapy measurable with dynamic liver function tests. This might help to optimize the chemotherapy-free interval prior to liver surgery and therefore needs to be addressed in a larger cohort.

## Consent

Written informed consent was obtained from both patients for publication of this Case Report and any accompanying images. A copy of the written consent is available for review by the Editor-in-Chief of this journal.

## Abbreviations

CALI: Chemotherapy associated liver injury
ICG: Indocyanine green
PDR: Plasma disappearance rate
ALT: Alanine transaminase
INR: International normalized ratio
BChE: Butyrylcholinesterase
